# Effects of Rice Straw Incorporation on Paddy Soil Microbiome and Metabolome Throughout the Crop Growth Period

**DOI:** 10.3390/microorganisms14061341

**Published:** 2026-06-15

**Authors:** Zhigang He, Bingshuang Wang, Dandan Jin, Muyu Tian, Liang Gong

**Affiliations:** Institute of Plant Nutrition and Environmental Resources, Liaoning Academy of Agricultural Sciences, Shenyang 110161, China; hezhigang1227@126.com (Z.H.); wangbingshuang2021@163.com (B.W.); jdd851228@sina.cn (D.J.); tian_muyu@163.com (M.T.)

**Keywords:** straw amendment, soil microbiota, metagenomic profiling, diversity

## Abstract

Rice straw incorporation is a paddy soil management practice that can reduce environmental pollution, mitigate soil degradation, and minimize nutrient loss. In this study, temporal shifts in soil microbial communities and metabolic profiles were investigated across three key rice growth stages—pre-planting (BS), tillering (TI), and harvest (HA)—to elucidate the ecological effects of straw incorporation. The Shannon diversity and Pielou evenness indices were significantly higher under straw incorporation than under the control at the BS and TI stages, but significantly lower at the HA stage. Straw incorporation also increased the relative abundance of key bacterial taxa, including *Polaromonas* sp. AER18D145, *Sphingomonas sediminicola*, and *Thiobacillus denitrificans*. Functional annotation indicated that the microbial community was mainly associated with amino acid biosynthesis and glycolysis. Metabolomic analysis revealed significant changes in steroids and their derivatives, terpenoid lipids, and carboxylic acids and their derivatives. Three metabolites—3-hexa-isoprenyl-4,5-dihydroxybenzoic acid, LysoPE (16:1(9Z)/0:0), and stachyose—differed significantly across all stages, suggesting their potential as metabolic indicators of straw incorporation. KEGG enrichment analysis identified significant alterations in arachidonic acid, purine, galactose, and pyrimidine metabolism. Redundancy analysis further revealed positive associations of LysoPE (16:1(9Z)/0:0) and stachyose with *Brevundimonas* sp. Root608 and *Polaromonas* sp. AER18D145.

## 1. Introduction

The incorporation of rice straw into paddy fields as a component of sustainable agricultural development confers ecological benefits by improving soil nutrient cycling and microbial functionality. In the major rice-producing regions of China, several hundred million tons of straw are generated annually, and straw incorporation has become a mainstream approach for its resource utilization. This practice not only promotes the recycling of agricultural waste but also plays a critical role in ensuring food security [1]. The degradation of straw facilitates the succession of soil microbial communities, thereby enhancing the expression and regulation of functional genes. This process enriches essential elements such as carbon (C), nitrogen (N), and phosphorus (P) in the soil, thus improving soil resources for crop production. Therefore, systematically characterizing the temporal dynamics of these metabolites is a prerequisite for elucidating the intrinsic link between rice straw decomposition and soil productivity. Studies have shown that straw input significantly alters the soil carbon-to-nitrogen ratio (C/N), providing microorganisms with abundant carbon sources and energy substrates and thereby driving a rapid succession of the microbial community [2]. However, the majority of existing studies have focused on specific time points, lacking a comprehensive analysis of microbial and metabolic networks across the entire straw return cycle, including the pre-planting, tillering, and harvest stages. This temporal fragmentation limits our understanding of the dynamic interplay between straw decomposition and soil functionality. Consequently, how microbial communities and their metabolic functions orchestrate the transformation of organic matter across distinct crop growth phases remains poorly understood.

The incorporation of rice straw into the soil creates a favorable habitat for microorganisms, leading to changes in soil microbial communities, such as increased bacterial diversity and microbial functional gene abundance [3]. The plant rhizosphere microhabitat is the most active microhabitat in soil and is also the main area for plants to obtain nutrients. In this microdomain, plant–microbial–soil–environment interactions collectively sustain the equilibrium of the rhizosphere microecosystem and affect crop production. Plant-associated microbes are crucial for host nutrient utilization, stress tolerance, plant health, and adaptation [4,5]. The majority of existing studies primarily emphasize the metagenomic analysis of single-time-point samples. While these studies elucidate changes in microbial metabolic functions, they tend to overlook the dynamic fluctuations in the soil’s intrinsic metabolome [6]. Over different cultivation periods, key microbial metabolites in the soil, including cellulose, hemicellulose, amino acids, and short-chain fatty acids, exhibit substantial variations [7]. In recent years, advances in multi-omics technologies have provided new insights into the microbial metabolic mechanisms underlying straw incorporation. Using a multi-omics approach, Zhang et al. found that nitrogen application, during the early stage of straw decomposition, shaped a microbial community centered on Bacillus and Staphylococcus [8]. Notably, although Staphylococcus lacked direct decomposing capacity, it restructured the Bacillus-dominated community with strong decomposition potential through competition for carbohydrate nutrients, thereby increasing the straw decomposition rate by 17%. Similarly, Lu et al. reported that the lipid metabolic network of the microbial community in paddy soil could be reprogrammed within a few days during drainage, with triglycerides being the most responsive class of metabolites [9]. Furthermore, Wang et al. demonstrated that under salt stress, both rhizosphere bacterial diversity and metabolite composition changed significantly, with lipids and lipid-related metabolites being upregulated; they also isolated rhizosphere plant-growth-promoting bacteria that enhanced rice salt tolerance [10]. Therefore, systematically characterizing the temporal dynamics of these metabolites is a necessary prerequisite for uncovering the intrinsic link between straw decomposition and soil productivity. This study systematically investigated key growth stages of rice (pre-planting, tillering, and harvest) and integrated metagenomics with non-targeted metabolomics analysis, thereby comprehensively evaluating the impacts of rice straw incorporation on soil microecology. This research offers not only multi-omics evidence for the resourceful utilization of straw but also valuable insights into the precise regulation of soil microbial functions.

## 2. Materials and Methods

### 2.1. Materials and Experimental Design

The pilot area was located in Panshan County, Liaoning Province, China (122°14′1″ E, 41°9′31″ N), within the location test area for the return of saline-alkali soil rice straw of the Liaoning Academy of Agricultural Sciences. The region belongs to the temperate subhumid continental monsoon climate, with an average annual temperature of 8.3 °C, an average annual precipitation of 624 mm, and a frost-free period of 172 days. The long-term rice straw-returning field experiment was established in 2019, and soil samples for this study were collected in 2024 after five years of continuous positioning. The soil type was coastal saline paddy soil, and the physical and chemical properties of the topsoil (0–20 cm) were as follows: pH of 6.96, organic matter content of 29.75 g·kg^−1^, total nitrogen content of 1.42 g·kg^−1^, total phosphorus content of 0.59 g·kg^−1^, available phosphorus content of 12.65 mg·kg^−1^, and available potassium content of 201.95 mg·kg^−1^ [11]. Coastal saline-alkaline soil, as a distinctive product of land–sea interactions, is formed through the combined effects of geological sedimentation and hydrological processes. Sediments and salts transported by marine tides are deposited along coastal margins, and because the rate of salt accumulation exceeds the rate of soil formation, these soils exhibit typical limiting characteristics, including high salinity, alkalinity, low fertility, and degraded structure [12].

The nitrogen fertilizer was urea (N 46%), with an application amount of N 260 kg·hm^−2^. The phosphate fertilizer was diammonium phosphate (N 18%, P_2_O_5_ 46%), and the application rate was P_2_O_5_ 90 kg·hm^−2^. The potassium fertilizer was potassium chloride (K_2_O 60%), and the application amount was K_2_O 90 kg·hm^−2^. Among them, N fertilizer was applied three times (6:3:1), including base, tillering, and ear fertilizer. Phosphate and potassium fertilizers were applied as base fertilizers in full amounts at one time. The fertilizer dosage of the N treatment was the same as that of the C treatment. The amount of straw returned to the field was 10.5 t·hm^−2^, which was basically the full amount of straw returned to the field. After being crushed, the rice straw was incorporated into the soil at a depth of 15 cm below the surface. Conventional field management practices were followed throughout the study.

The rice variety tested was ‘Yan Feng 47’, which is a medium-late japonica rice cultivar bred by the Liaoning Institute of Saline-Alkali Land Utilization. It is well known for its saline-alkaline tolerance, high yield, and good grain quality. With tolerance to salinity levels of 0.3–0.4% and up to pH 9.0, Yanfeng 47 has become the preferred japonica rice variety for saline-alkaline soils in Liaoning. Two treatments, each with three replicates, were established: chemical fertilizer (C) and straw return with chemical fertilizer application (N). Each test area measured 15 m^2^ (5 m × 3 m) and was separated by polyvinyl chloride (PVC) boards (PVC boards were inserted every 20 cm). The rice seedlings were transplanted to the field on 21 May 2024. The distance between each row was 30 cm. In each experimental area, six sampling plots were randomly established using the five-point sampling method, and soil samples were collected from each plot using the random five-point method. Samples were collected before planting (BS), during the tillering stage (TI), and after harvest (HA), totaling 18 samples. The samples were stored at −80 °C until testing.

The QIAGEN DNeasy Mericon Food Kit DNA genome extraction kit was purchased from QIAGEN, Hilden, Germany; the Illumina Novaseq 6000 high-throughput sequencing platform reagents were sourced from Illumina, San Diego, CA, USA; methanol, acetonitrile, formic acid, and ammonia water were obtained from Fisher Scientific, Waltham, MA, USA; ultrapure water was supplied by Watsons Group Limited, Hong Kong; and ACQUITY UPLC HSS T3 C18 (2.1 × 100 mm, 1.8 μm) was provided by Waters, Milford, MA, USA.

### 2.2. Instruments and Equipment

An Illumina Xten high-throughput sequencing platform was purchased from Illumina, Inc. in the San Diego, CA, USA; an R950 rack-mounted server was purchased from Dell, Inc. in the Round Rock, TX, USA; a JXFSTPRP-64 fully automatic rapid-sample-grinding instrument was purchased from Shanghai Jingxin Industrial Development Co., Ltd., Shanghai, China; an SCIEX 6600 UPLC-Q-TOF-MS was purchased from the Danaher Corporation Group in the Washington, DC, USA; an IND-2000C micro-UV spectrophotometer was purchased from Nano Drop in the Wilmington, DE, USA; and a Heraeus Multifuge X3R low-temperature centrifuge was purchased from Thermo Fisher Scientific in the Waltham, MA, USA.

### 2.3. Empirical Method

#### 2.3.1. Pre-Treatment of Metagenomic Samples

Approximately 2.0 g of the soil samples was transferred into a 5 mL sterile, enzyme-free centrifuge tube. The microbial metagenomic DNA was extracted from the soil sample using the protocol provided in the QIAGEN DNeasy Mericon Food Kit DNA Genome Extraction Kit (Qiagen, Hilden, Germany). The concentration and purity of the DNA were analyzed using a UV spectrophotometer and via agarose gel electrophoresis. Qualified DNA was fragmented to approximately 350 bp using a Covaris M220 ultrasonicator (Covaris, Woburn, MA, USA), and paired-end (PE) libraries were constructed using the NEXTFLEX Rapid DNA-Seq Kit (Bioo Scientific, Austin, TX, USA). The qualified DNA libraries were then subjected to metagenomic sequencing on the Illumina X Ten platform. Raw sequencing data were quality-controlled using fastp software (v0.20.0) [13].

#### 2.3.2. Bioinformatics Analysis

The soil metagenomic data were subjected to quality control and rice genome sequence removal using KneadData software (Version 0.12.2), and the high-quality metagenomic data were used for subsequent analysis [14]. This study utilized the HMP Unified Metabolic Analysis Network (HUMAnN 3) analysis process to analyze the quality-controlled metagenomic data [15]. kraken2 software (Version 2.1.3) was used to parse the microbial community structure information in the soil [16], and the metagenomic data were analyzed for microbial functional characteristics and metabolic pathways based on UniRef 90 data. R software was used to analyze the microbial α diversity index and β diversity.

#### 2.3.3. Pre-Treatment of Metabolomic Samples

Following the method modified by Bell (2022) [17], 1.0 g of the soil sample was accurately weighed, 2 mL of 50% methanol water solution was added, and 3 zirconia beads were included. The mixture was pre-cooled at −20 °C for 10 min and then placed in a grinder, where it was ground for 1 min at 60 Hz. This process was repeated. The homogenized sample was then transferred to a 5 mL centrifuge tube and centrifuged at 10,000× *g* for 10 min. A total of 1 mL of the supernatant was transferred to a 2 mL centrifuge tube, and this step was repeated once. The sample was filtered through a 0.22 um organic filter membrane into an LC injection bottle until testing. The quality control (QC) sample was prepared by mixing all samples, with the order of samples randomized. Before the analysis run, the QC sample was injected five times to assess system stability, as well as between each five analysis runs.

#### 2.3.4. Test Conditions

Non-targeted metabolomics analysis was performed using an ultra-high-performance liquid chromatography (UPLC) system equipped with a quadrupole time-of-flight mass spectrometer. Chromatographic separation was carried out at 25 °C, with a flow rate of 0.4 mL/min and an injection volume of 2 μL. The mobile phase for the cationic mode consisted of 0.1% formic acid in Watson’s water (A) and 0.1% formic acid in acetonitrile (B). For the negative ion mode, the mobile phase comprised 0.1% ammonia in Watson’s water (A) and acetonitrile (B). The gradient elution program was as follows: 0–0.5 min 5% B, 0.5–3, 0–5 min 5–20% B, 3–5 min 20–50% B, 5–9 min 50–70% B, 9–10 min 70–95% B, 10–13 min 95–95% B, 13–15 min 95–5% B. Nitrogen served as the carrier gas. The conditions for the electrospray interface were as follows: ion source gas 1 (60 psi), ion source gas 2 (60 psi), ion source temperature 600 °C, deionization voltage (±50 V), and collision voltage (30 ± 10 V). For the negative and positive ion modes, the fragmentation voltages were set to −4500 V and 5500 V, respectively. In full-scan resolution mode, the mass range was set from 50 to 1000 Da.

#### 2.3.5. Metabolite Identification

The raw data were imported into Progenesis QI software (Version 4.0) (Waters Company, USA) to obtain a data matrix that includes retention times, mass-to-charge ratios, and peak intensities. Metabolites were identified by searching HMDB (http://www.hmdb.ca/ accessed on 23 December 2024), Metlin (https://metlin.scripps.edu/ accessed on 23 December 2024), and the internal database. The matrix data were analyzed, and these metabolites were classified using HMDB. Pathway enrichment analysis was conducted using the Kyoto Encyclopedia of Genes and Genomes (KEGG) database.

### 2.4. Data Analysis and Visualization

In this study, R software was used to analyze the relative abundance of microorganisms in soil, α diversity index, and differences in microbial metabolic pathways, and principal coordinate analysis (PCoA) was performed on the microbial community structure [18]. Multivariate statistical analysis, including principal component analysis (PCA) and partial least squares discriminant analysis (PLS-DA), was performed using Metaboanalyst 6.0 online website, and T-test results, variation ratios, and drawings were analyzed using R (4.3.2) software. The screening conditions for differential metabolites were set as VIP > 1.0, *p* < 0.05, and FC ≥ 2 or ≤0.5. All data analyses and visualizations were completed in R software and Origin 2021.

## 3. Results

### 3.1. Soil pH and Organic Carbon

**Figure 1:**
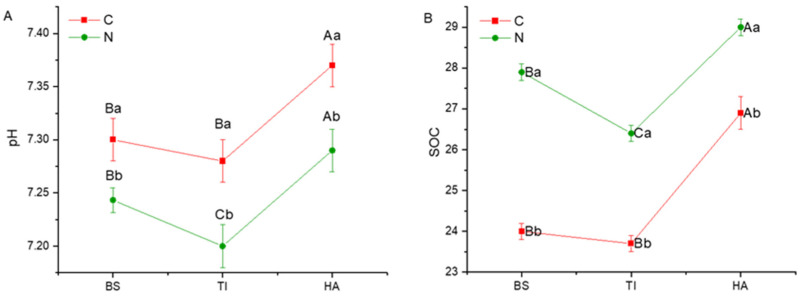
Effects of straw addition on soil pH (**A**) and SOC (**B**) at different stages. Note: BS: pre-planting; TI: tillering; HA: harvest. Different uppercase letters indicate significant differences among stages within the same treatment, whereas different lowercase letters indicate significant differences between treatments within the same stage.

Soil pH exhibited significant stage-dependent variation across different growth stages. In the CK treatment, soil pH after harvest was significantly higher than that before sowing and at the tillering stage. In the straw incorporation treatment, soil pH after harvest was significantly higher than that before sowing, and the pH values before sowing and after harvest were both significantly higher than those at the tillering stage. Comparisons between treatments showed that soil pH in the straw incorporation treatment was lower than that in the control at all three stages, namely before sowing, during tillering, and after harvest, indicating that straw incorporation maintained a lower soil pH level throughout the entire growing period (Figure 1A).

Soil organic carbon also showed significant stage-dependent variation across different growth stages. Overall, soil organic carbon content in all treatments was at a moderate to high level before sowing, decreased slightly at the tillering stage, and increased significantly after harvest. Comparisons between treatments further revealed that soil organic carbon content in the straw incorporation treatment was significantly higher than that in the control at all three stages, including before sowing, during tillering, and after harvest, and this difference remained stable throughout the observation period (Figure 1B).

### 3.2. Microbial Diversity in Soil Samples

**Figure 2:**
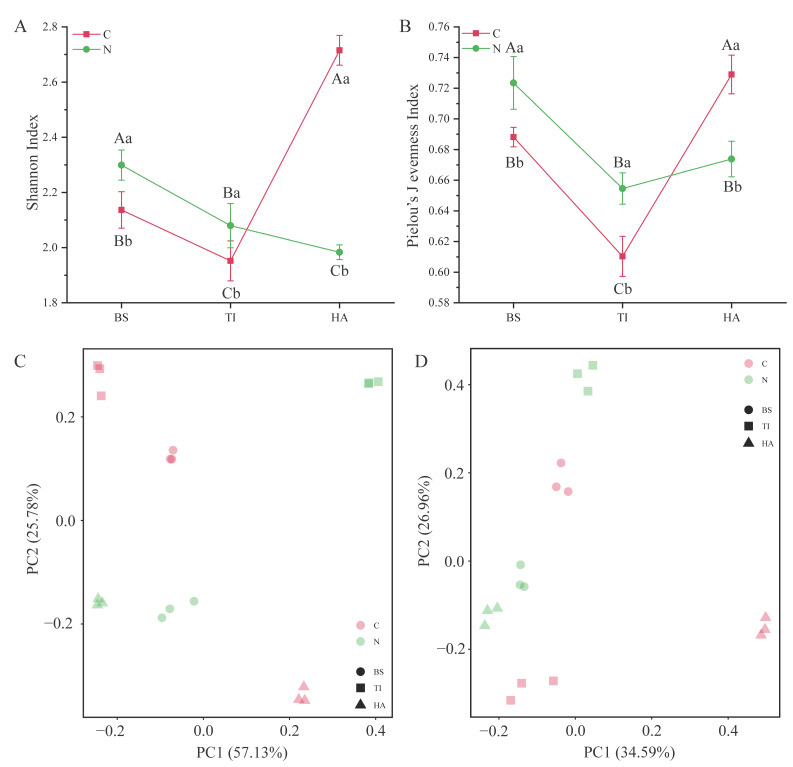
Microbial diversity analysis of straw incorporation (N) versus control (C) groups. Note: (**A**,**B**) denote the α diversity index of all samples; (**C**,**D**) represent the PCoA of all samples. Rice growth stages—BS: pre-planting; TI: tillering; HA: harvest. Different uppercase letters indicate significant differences among different stages within the same treatment, whereas different lowercase letters indicate significant differences among different treatments within the same stage.

Metagenomic analysis, owing to its unique technical advantages, has a natural advantage in analyzing microbial diversity in environmental samples. As illustrated in Figure 2A,B, the Shannon and J indices of microorganisms in Group N were significantly higher than those in Group C during the BS and TI periods (*p* < 0.05), but significantly lower during the HA period. Notably, the diversity and richness of microorganisms in Group N showed a significant decline, while those in Group C increased significantly later on (*p* < 0.05). As shown in Figure 2C,D, the principal coordinate analysis based on Bray–Curtis distance indicates a clear separation trend between Groups C and N in two-dimensional space. Additionally, the study found that samples from different periods of Group C showed a more pronounced separation trend, while those from Group N were relatively close during the BS and HA periods, and more distant from those during the TI period. This suggests that rice straw incorporation into the soil significantly alters the overall structure and diversity of soil microorganisms, potentially maintaining their stability.

### 3.3. Analysis of Microbial Composition and Variation in Soil Samples

**Figure 3:**
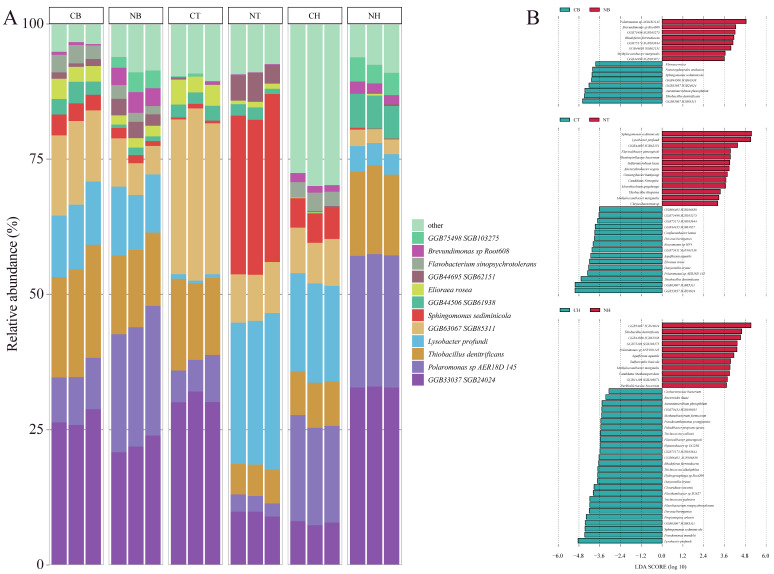
Microbial composition analysis of straw incorporation (N) versus control (C) groups. Note: (**A**,**B**) denote the species composition of all samples; (**B**) indicates different species. CB: control treatment before sowing; NB: straw application treatment before sowing; CT: control treatment at the tillering stage; NT: straw application treatment at the tillering stage; CH: control treatment after harvest; NH: straw application treatment after harvest.

Based on the analysis of the overall structure and diversity of microorganisms in different groups, this study further analyzed the microbial composition and differential microorganisms in the samples. As shown in Figure 3A, the dominant bacterial species (with an average relative content exceeding 1%) in the soil samples include GGB33037 SGB24024, *Polaromonas* sp. AER18D145, *Thiobacillus denitrificans*, *Lysobacter profundi*, and *Sphingomonas sediminicola*, with average relative contents of 21.69%, 14.00%, 13.13%, 12.17%, and 6.80%, respectively.

Meanwhile, this study further analyzed the differences in microorganisms between the two soil groups at different times. The results showed that during the BS period, the abundance of *Polaromonas* sp. AER18D145, *Brevundimonas* sp. Root608, *Rhodoferax ferrireducens*, and *Methyloceanibacter marginalis* were significantly higher in Group N (*p* < 0.05). During the TI period, the abundance of *Sphingomonas sediminicola*, *Lysobacter profundi*, *Flavisolibacter ginsengisoli*, and *Rhodospirillaceae* bacteria was significantly higher in Group N (*p* < 0.05). During the HA period, the abundance of *Thiobacillus denitrificans*, *Polaromonas* sp. AER18D145, *Aquiflexum aquatile*, and *Sulfuricaulis limicola* were significantly higher in Group N (*p* < 0.05). These findings indicate that rice straw incorporation has a great influence on the structure of microorganisms in the soil and mainly increases the abundance of *Polaromonas* sp. AER18D145, *Sphingomonas sediminicola* and *Thiobacillus denitrificans* in the soil.

### 3.4. Functional Analysis of Microorganisms in Soil Samples

Microbial community functions play a central role in soil ecosystems, impacting soil health maintenance, nutrient cycling, pollutant degradation, and crop productivity enhancement. Therefore, this study further examined the functional aspects of microorganisms in soil at different times, based on the analysis of microbial community structure.

**Figure 4:**
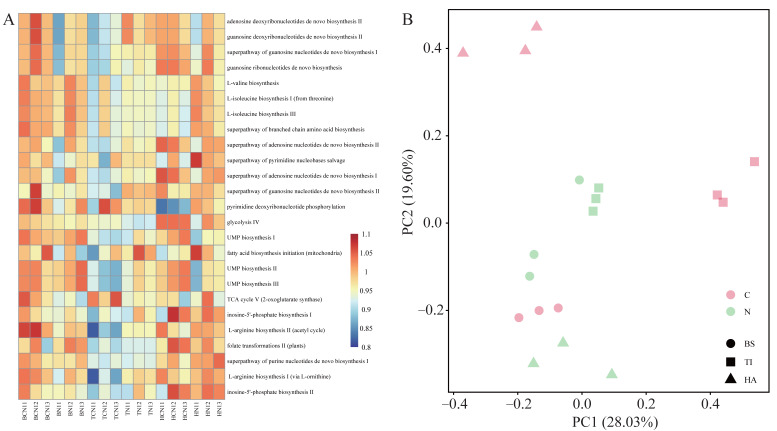
Microbial function analysis of straw incorporation (N) versus control (C) groups. Note: (**A**) shows the main metabolic pathway of all samples; (**B**) shows the PCA results of all samples.

In Figure 4A, the primary functional genes of microorganisms in the soil are annotated for adenosine deoxyribonucleotides and ornithine from scratch biosynthesis, L-lysine biosynthesis, amino acid biosynthesis including L-isoleucine, and glycolysis, among other energy metabolism pathways. Notably, Groups C and N show a clear separation trend in two-dimensional space. Additionally, samples from different periods of Group C exhibited a more pronounced separation trend, while Group N remained relatively close across all time points.

### 3.5. Quality Control, PCA, and PLS-DA

**Figure 5:**
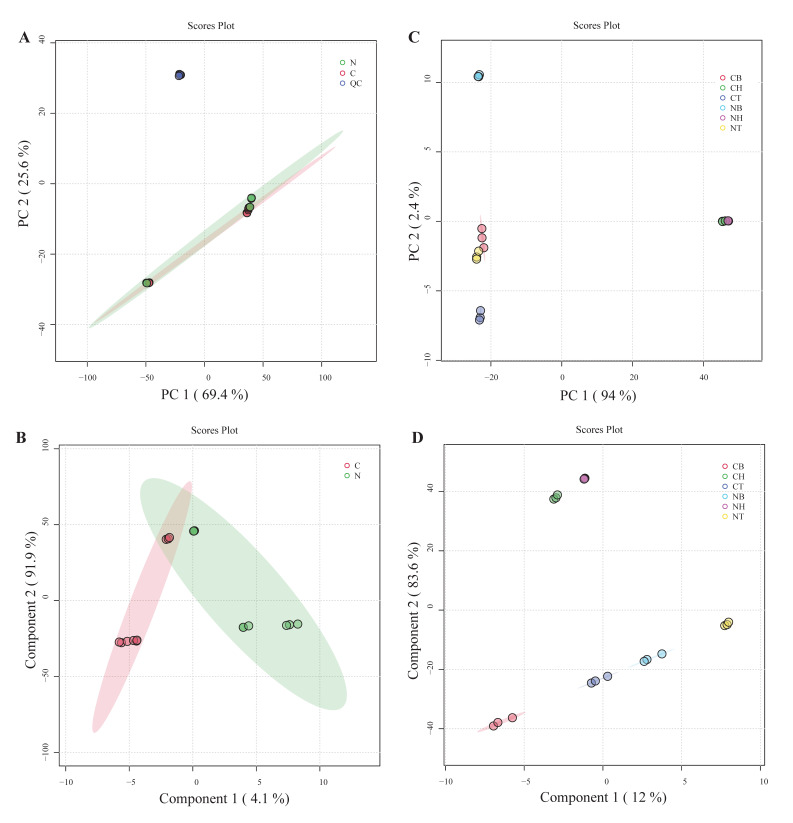
PCA and PLS-DA plots of straw incorporation (N) versus control (C) groups. Note: (**A**,**C**) show the PCA results of all samples; (**B**,**D**) show the PLS-DA results of all samples. Microbial function analysis of straw incorporation (N) versus control (C) groups.

The QC samples were used to evaluate the testing stability and repeatability of the instrument. As shown in Figure 5A, all QC samples were clustered together, indicating that the instrumental system stability was maintained during this test, providing precise and reliable data for subsequent experiments. First, PCA and PLS-DA were conducted on soil samples from different periods of rice cultivation, as illustrated in Figure 5. Substantial overlap was observed between Groups C and N; however, a separation trend was also evident among soil samples from different time points. Subsequently, PCA and PLS-DA were performed on samples from different time points, revealing a distinct separation trend among the samples from each group. Both time points and the incorporation of rice straw into the paddy field exerted significant influence on the soil’s metabolic profile. The two groups of samples before planting were concentrated in the third quadrant, while the soil samples at harvest time were clustered in the fourth quadrant (Figure 5C), with a high degree of overlap in the metabolic profiles of the two groups. In Figure 5D, the distribution trends of metabolites in each group also showed similar patterns.

### 3.6. OPLS-DA and Differential Metabolite Screening of Soil Samples

**Figure 6:**
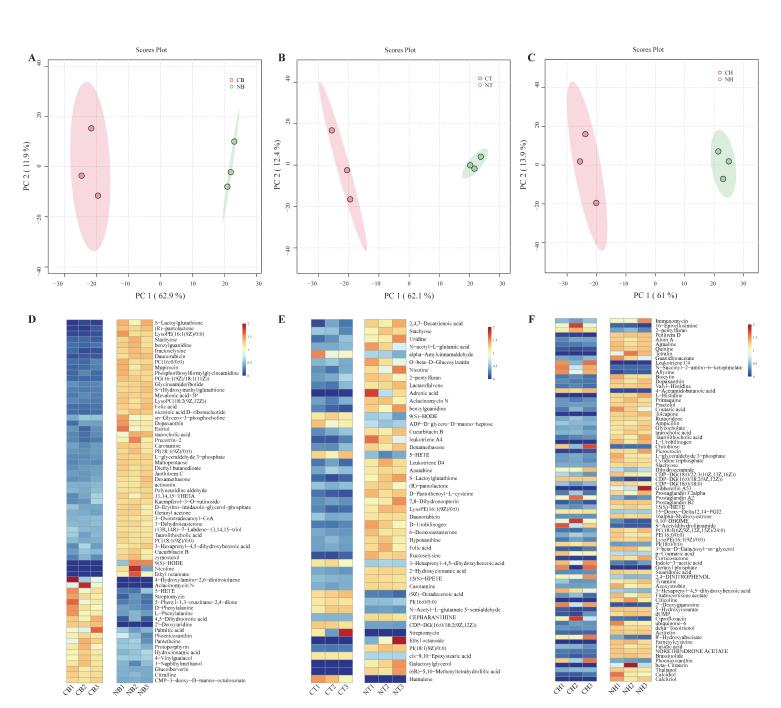
Differential metabolites induced by straw incorporation across time periods. Note: (**A**–**C**) show the OPLS-DA maps before planting, tillering, and harvest, and (**D**–**F**) show the heat maps of differential metabolites at different times.

The PCA and PLS-DA plots above show that soil samples from the same time point exhibited more similar metabolic profiles, indicating that the incorporation of rice straw into the paddy field has a time-dependent regulatory effect on soil metabolism. Subsequently, OPLS-DA was conducted on soil samples from different periods, revealing a significant separation trend in both groups of samples, indicating that the incorporation of rice straw into the paddy field significantly alters the soil’s metabolic profile. Using VIP, P, and FC as criteria, 65, 47, and 78 differential metabolites were identified before planting, during the tillering stage, and at harvest, respectively. Classification of these metabolites using the HMDB database revealed that the incorporation of rice straw into the paddy field has a similar impact on soil metabolites across different periods, with these metabolites primarily concentrated in steroid compounds and their derivatives, terpenoid lipids, carboxylic acids and their derivatives, fatty acyl compounds, and glycerophospholipids (Figure 6).

### 3.7. Metabolic Pathway Enrichment Analysis of Soil Samples

**Figure 7:**
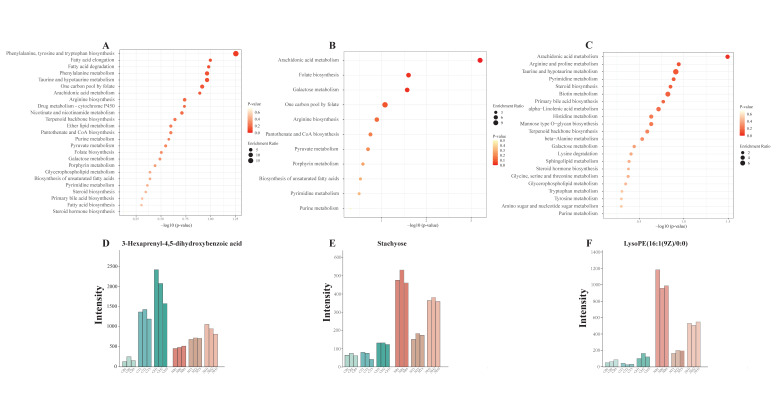
Differential metabolites and enriched pathways during straw incorporation at different time points. Note: (**A**–**C**) show the KEGG pathway enrichment maps of differential metabolites before planting, tillering, and harvest (the color indicates the *p* value, and bubble size represents the pathway impact factor). (**D**–**F**) show the relative abundances of 3-Hexaprenyl-4,5-dihydroxybenzoic acid, Stachyose, and LysoPE(16:1(9Z)/0:0).

By conducting pathway enrichment analysis on differential metabolites from soil samples at three time points, the impact of rice straw incorporation on the metabolic pathways of the samples could be compared. Before planting, the incorporation of rice straw into the paddy field led to the enrichment of metabolic pathways in amino acid metabolism (phenylalanine metabolism, arginine biosynthesis, etc.), lipid metabolism (fatty acid degradation, steroid biosynthesis, etc.), energy and carbon metabolism (pyruvate metabolism, galactose metabolism), vitamin and cofactor metabolism (folate biosynthesis, niacin and nicotinamide metabolism), and nucleotide metabolism (purine metabolism, pyrimidine metabolism). During the tillering stage, 11 metabolic pathways were enriched, including arachidonic acid metabolism, folate biosynthesis, galactose metabolism, and arginine biosynthesis. At harvest, 22 pathways were enriched, including lipid metabolism, amino acid metabolism, carbohydrate metabolism, nucleotide metabolism, and vitamin and cofactor metabolism. The results of the metabolic pathway analysis indicate that the treatment of incorporating rice straw into the paddy field primarily regulates four metabolic pathways: arachidonic acid metabolism, purine metabolism, galactose metabolism, and pyrimidine metabolism, all of which showed significant changes across different treatment periods (Figure 7).

### 3.8. Multi-Omics Integrated Analysis

**Figure 8:**
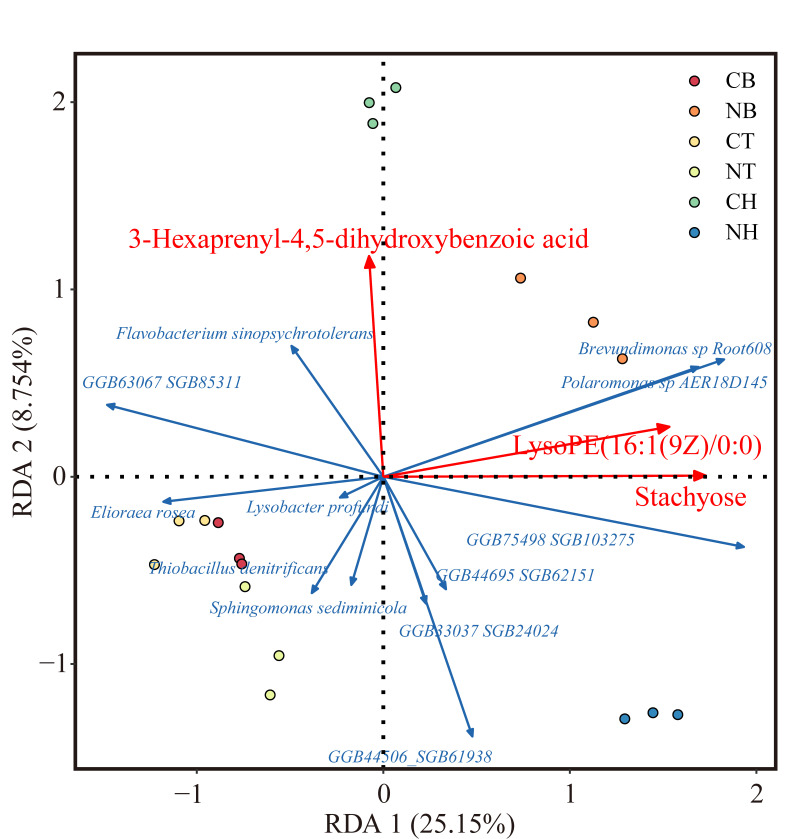
Redundancy analysis of the association between metagenomics and differential metabolites at different stages of rice straw incorporation and returning to the field.

To gain a deeper understanding of the core aspects of soil ecosystem functions and regulatory mechanisms, it is essential to employ multi-omics integrated analysis. Redundancy analysis (RDA) further revealed significant associations between key microorganisms and differential metabolites. The results showed that RDA1 and RDA2 explained 25.15% and 8.75% of the total variation, respectively, accounting for 33.90% of the variation in total. Lysophosphatidylethanolamine (16:1(9Z)/0:0) and stachyose showed a strong positive correlation with *Brevundimonas* sp. Root608 and *Polaromonas* sp. AER18D145, as well as a strong negative correlation with Rosea daylily bacterium (*Elioraea rosea*). Additionally, 3-hexa-isoalliene-4,5-dihydroxybenzoic acid showed a strong positive correlation with *Flavobacterium sinopsychrotolerans*, as well as a strong negative correlation with *Sphingomonas sediminicola* and *Thiobacillus denitrificans* (Figure 8).

## 4. Discussion

### 4.1. Effects of Rice Straw Return to the Field on Soil Metagenome

Rice straw incorporation refers to the mechanical or manual burial of straw into the soil, aiming to increase organic matter content and enhance soil fertility [19]. As an effective soil improvement measure, it can elevate the carbon and nitrogen content and microbial diversity of paddy fields, thereby enhancing rice growth and yield [19]. Research indicates that returning straw to the field significantly influences the composition and diversity of soil microorganisms. It not only effectively alleviates carbon and nitrogen limitations for microorganisms, thereby enhancing nutrient cycling efficiency, but also improves soil pore structure through incorporation, increasing microbial biomass and enzyme activity [20]. This study found that incorporating rice straw into the soil can significantly enhance the diversity and richness of soil microorganisms, as well as improve their composition. The incorporation of rice straw into the soil provides a substantial source of organic matter, thereby facilitating the proliferation of heterotrophic microorganisms [2]. Additionally, the decomposition of straw releases various small organic molecules, such as sugars, organic acids, and phenolic compounds, which serve as energy sources and metabolic substrates for microorganisms, promoting an increase in soil microbial numbers and changes in community structure [21]. Interestingly, this study found that the Shannon and evenness indices of the microbial community in the N group were significantly higher than those in the C group during the BS and TI stages (*p* < 0.05) but were significantly lower than those in the C group during the HA stage, showing a dynamic pattern of “first increasing and then decreasing.” This result may indicate that the effect of straw application on soil microbial diversity is stage-dependent. At the early stage of straw input, the addition of exogenous organic matter stimulated the rapid proliferation of microorganisms, resulting in a significant increase in diversity; however, at the later stage of crop growth, diversity declined with the depletion of easily degradable organic matter and environmental changes associated with crop maturation. Compared with other studies on paddy soils, the key genera identified in this study showed good generality. Iniesta-Pallarés et al. found that *Thiobacillus* was highly enriched in non-rhizosphere soil in rice fields, and this genus was a core participant in sulfur cycling in paddy soils [22]. This is consistent with the results of this study, indicating that the abundance of *Thiobacillus denitrificans* increased significantly during the HA stage. In addition, previous studies have shown that Proteobacteria accounted for more than 50% of the active microorganisms in flooded paddy soils, which is in agreement with the significantly increased abundances of *Polaromonas* sp. *AER18D145* and *Sphingomonas sediminicola* observed in this study [23]. Furthermore, studies have shown that after incorporating rice straw into the soil at different times, the abundance of *Polaromonas* sp. AER18D145, *Sphingomonas sediminicola*, and *Thiobacillus denitrificans* are higher. *Polaromonas* is a genus of bacteria widely distributed in soil, freshwater, and sediment environments. This genus has the metabolic potential to degrade a variety of aromatic compounds, such as benzoates, phenols, polycyclic aromatic hydrocarbons, and complex organic polymers [24]. Research has confirmed that *Polaromonas* exhibits strong metabolic activity, particularly in the decomposition of organic matter. Its proliferation accelerates the mineralization process of refractory components in straw, thus speeding up the turnover of soil organic carbon and increasing the rate of nutrient release from the soil, enhancing the turnover of soil organic carbon and increasing the rate of nutrient release for crop root growth [25]. *Sphingomonas sediminicola* has a strong ability to degrade aromatic compounds, which positively impacts the degradation and purification of pesticides in farmland and the rhizosphere environment. *Thiobacillus denitrificans* promotes crop growth by enhancing sulfur supply, regulating nitrogen cycling, and improving soil pH. As an environmentally responsive functional bacterium, it reflects the restoration and enhancement of soil ecological functions, providing a crucial microbial foundation for sustainable agricultural development [26].

### 4.2. The Impact of Rice Straw Return to the Field on Soil Metabolome

Changes in the nutrients and microorganisms in the soil can lead to changes in metabolites. Before planting, the incorporation of rice straw into the soil resulted in a significant increase in 48 types of metabolites. During the tillering stage, 13 types of metabolites decreased, while at harvest time, 55 types of metabolites increased. This indicates that the incorporation of rice straw significantly boosted the levels of most differential metabolites in the soil. According to the classification results, the incorporation of rice straw increased the levels of fatty acyl compounds, steroidal compounds and their derivatives, carboxylic acids and their derivatives, and terpenoid lipids. Chang (2015) [27] found that green manure can enhance the levels of amino acid substances and carboxylic acid substances in the soil, which is consistent with the findings of this study. The incorporation of rice straw into the soil increases the levels of N, P, K, and trace elements, reducing reliance on chemical fertilizers. Additionally, the increased levels of carboxylic acids and other substances in the soil can improve crop yields and quality [28]. By conducting an intersection analysis of the differential metabolites induced by the incorporation of rice straw into the soil at various times, three common substances were identified: 3-hexa-pentadienyl-4,5-dihydroxybenzoic acid, hemolysin phosphatidylethanolamine (16:1(9Z)/0:0), and stachyose. These substances show significant changes across different periods and can serve as key differential metabolites in the incorporation of rice straw into the soil. Stachyose, a tetrasaccharide found in the seeds or roots of leguminous plants, acts as a carbon storage material that provides energy for crop growth and development [29]. In this experiment, the incorporation of rice straw into the soil significantly increased the production of stachyose. This is attributed to the high content of pectin and hemicellulose in the straw, which gradually degrade into monosaccharides such as glucose and lactose and are subsequently converted by microorganisms into stachyose and galactose [30].

The results of metabolic pathway analysis indicated that incorporating rice straw into the soil stimulates the production of substantial amounts of sugars and influences associated metabolic pathways. This enhances the soil’s capacity to generate additional carbon and nitrogen sources, thereby providing stable nutrients for crops and reducing dependence on chemical fertilizers.

Four metabolic pathways showed significant changes before planting, during tillering, and at harvest, indicating they can serve as core differential metabolic pathways based on the enrichment analysis of the differential metabolites resulting from the incorporation of rice straw into the soil. Liu (2022) [7] found that incorporating straw into the soil can improve soil quality metabolism, promoting the enrichment of pathways such as arachidonic acid, fatty acid synthesis, and glycerophospholipid metabolism. This finding aligns with our results, suggesting that incorporating rice straw into the soil helps improve soil quality and further promotes crop growth and development. Wu (2022) [31] discovered that incorporating straw into the soil significantly promotes the enrichment of pyrimidine and purine metabolic pathways in the soil. Straw, as an organic matter, gradually decomposes into small molecules, which are used by soil microorganisms as carbon and nitrogen sources, converting them into purines and pyrimidines, thus aiding in the renewal and replacement of nutrients in the soil [32]. Compared with other metabolomic studies on paddy soils, Lu et al. (2025) found that the lipid metabolic network in paddy soil was rapidly remodeled during drainage, with triacylglycerols being the most sensitive metabolites [9], which is consistent with the significant changes in lipid metabolites such as LysoPE observed in this study. However, this study further identified significant enrichment of the arachidonic acid metabolism pathway, which may be related to osmotic regulation under saline-alkaline stress. In addition, Cai Yuanfeng et al. (2014) reported that carbohydrate metabolism was the dominant metabolic process in conventional flooded paddy soils [23], whereas both lipid metabolism and amino acid metabolism pathways were enriched in the saline-alkaline paddy soils of this study, suggesting that under saline-alkaline conditions, microorganisms may require coordinated responses through multiple metabolic pathways to cope with straw input.

Straw returns to the field drives soil microbial-mediated biogeochemical processes, and has a significant impact on soil ecological functions. This study found that in straw return systems, the key functional bacterial group *Polaromonas* sp. AER18D145 plays a key role in degrading straw, facilitating the breakdown of cellulose and hemicellulose into small molecules such as monosaccharides, thereby promoting straw decomposition and nutrient release [25]. These degradation products can be absorbed and utilized by crops to synthesize osmoregulatory substances such as stachyose, thereby enhancing crop stress resistance. Concurrently, the accumulation of lysophosphatidylethanolamine (16:1(9Z)/0:0), a catabolic product of membrane phospholipids, directly reflects enhanced microbial biomass turnover and metabolic activity [33]. RDA further revealed significant associations between key microorganisms and differential metabolites: *Brevundimonas* sp. Root608 was positively correlated with LysoPE and stachyose. The genus *Brevundimonas* comprises bacteria with plant growth-promoting rhizobacteria (PGPR) potential, capable of producing indole-3-acetic acid (IAA), siderophores, and ACC deaminase, thereby promoting plant root development and alleviating stress [34]. This serves as a signal of the increased functional potential of the soil microbiome. Straw return also triggers dynamic succession in microbial community structure; rapidly proliferating decomposers (such as *Polaromonas*) may gain a niche advantage, thereby partially inhibiting the plant growth-promoting bacterium *Elioraea rosea*, which was originally active in the rhizosphere [35]. Furthermore, amino acid-metabolizing bacteria *Sphingomonas sediminicola* [36,37] and sulfur–nitrogen cycling bacteria *Thiobacillus* [38] participate in organic matter transformation and nutrient cycling by metabolizing 3-hexa-isoalliene-4,5-dihydroxybenzoic acid as a carbon or energy source, thereby reducing its concentration in the soil. Taken together, these findings reveal that straw incorporation not only directly drives organic matter decomposition and nutrient transformation but also, through environmental selection and interspecific interactions, guides microbial communities toward a new equilibrium. Ultimately, this process facilitates the efficient conversion of exogenous organic matter into endogenous soil fertility and leads to an overall enhancement of soil ecological functions.

## 5. Conclusions

This study systematically investigated the dynamic effects of rice straw incorporation on soil microbial and metabolic communities throughout the crop’s entire growth cycle. The results indicate that incorporating rice straw into the soil significantly enhances soil microbial diversity before planting and during the tillering stage, alters the composition and abundance of key functional bacterial groups, and stimulates the activity of metabolic pathways such as amino acid synthesis and glycolysis. Additionally, metabolic analysis revealed that incorporating rice straw induces changes in various bioactive metabolites, which further influence key metabolic pathways like arachidonic acid, purine, and galactose metabolism. Some metabolites, such as stachyose, are significantly associated with specific microorganisms, suggesting their potential role in microbial–metabolite interactions. In summary, incorporating rice straw into the soil, a sustainable agricultural practice, not only optimizes the soil microecological structure and function but also offers substantial ecological and practical benefits for achieving sustainable agricultural development.

## Data Availability

The metagenomic and metabolomic data from this study have been deposited into the National Genomics Data Center (NGDC) database (https://ngdc.cncb.ac.cn/ accessed on 25 March 2026) under accession number PRJCA059343.
